# Object symmetry effects in Germanic: Evidence for the role of case

**DOI:** 10.1007/s11049-018-9404-5

**Published:** 2018-03-06

**Authors:** Bill Haddican, Anders Holmberg

**Affiliations:** 1 CUNY-Queens College and Graduate Center, New York, United States; 2 Newcastle University, Newcastle, United Kingdom

**Keywords:** Passive, Double object construction, Passive symmetry, Locality, Case, Argument structure

## Abstract

This paper focuses on passive symmetry effects in Germanic. We describe two large-sample judgment experiments with native speakers of Norwegian and Swedish, two partially symmetric passive languages. The results fail to support predictions of Anagnostopoulou’s (2003) seminal locality approach to passive symmetry in these languages. We propose that constraints on object ordering in these varieties are better modeled on a revised version of classic case-based theories. On this approach, patterns of object ordering are governed by variation in the way that case is assigned to objects. In addition, the Norwegian results suggest a shape conservation effect in object shift contexts not previously reported in the literature. Theme-recipient orders in Norwegian object shift contexts are available for just those speakers who also accept theme-recipient orders in active non-object shift contexts. This object ordering constraint applies in the same environment that another, much better described ordering constraint applies, namely Holmberg’s Generalization effects. We show that these results are explained by Fox and Pesetsky’s (2005) cyclic linearization algorithm together with the assumption that theme-recipient orders vP-internally reflect short theme-movement above the recipient.

## Introduction

A central challenge for formal theories of argument movement is to model cross-linguistic variation in patterns of passivization out of double object constructions, a set of issues sometimes referred to as the “passive symmetry” problem. Work in comparative syntax dating from the late 1980s has revealed that languages with passive movement fall into one of two main classes with respect to passivization out of double object constructions. One class of languages—symmetric passive languages, including Norwegian, Kinyarwanda, Kinande, and some dialects of British English—has the property that, out of a double object construction, either object may passivize (Baker 1988; Doggett 2004; Georgala 2011a; McGinnis 1998, 2001; Ura 1996; Woolford 1993). We illustrate this in the Norwegian example in (1).

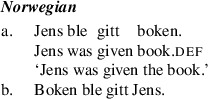

By contrast, “asymmetric passive languages” including Fula, Swahili, Chichewa, Danish, and many varieties of English, allow recipient arguments but not themes to passivize out of double object constructions (Baker 1988; Bresnan and Moshi 1990; Postal 2004; Woolford 1993). We illustrate this in the Danish example in (2).

(2)

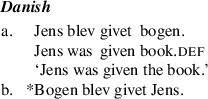

The challenge for formal theories posed by these facts is to explain the nature of the representational differences between double object passives in these two classes of languages. In the generative literature, two main approaches to these facts have been proposed. One approach prominent in the Government and Binding tradition explains this variation in terms of differences in the way that nouns are licensed via case in these two kinds of languages. A premise of this approach is that, in passive contexts, the objective case that would otherwise be assigned by the transitive verb is “absorbed” by passive morphology, and derived subjects move to subject position to receive case (Jaeggli 1986; Roberts 1987). On this approach, the symmetric pattern reflects the fact that case for either object may be absorbed, with the consequence that either object may passivize. In asymmetric passive languages, on the other hand, only the case that would otherwise be assigned to the recipient may be absorbed, with the consequence that only the recipient argument may passivize.

A second approach—the consensus in recent minimalist work—has explained these facts not in terms of case but rather locality. On this approach, what blocks theme passivization in asymmetric passive languages is the fact that the recipient argument intervenes between the thematic position of the theme and subject position. The relevant case- or category-sensitive dependency relation responsible for theme passivization cannot be established because of matching case or categorial features on the intervening recipient argument. What fixes this problem in symmetric passive languages is the availability of a short theme movement to an “equidistant” position—typically an outer specifier of the same projection hosting the recipient. A subsequent movement step raises the theme to subject position. Because this intermediate “stepping stone” position is in a projection with a segment immediately dominating the recipient argument, neither of these movement steps “crosses over” the recipient argument, and therefore no locality problem arises (Anagnostopoulou 2003; Doggett 2004; McGinnis 1998, 2001; Jeong 2007; Ura 1996). We illustrate this approach in (3).

(3) [TP Theme [XP Theme Recipient [YP Theme ] ] ]

A crucial empirical point on which the locality analysis turns is the availability of independent evidence of the short theme movement in (3). Anagnostopoulou (2003) proposes that evidence to this effect comes from patterns of object ordering in passive and active object shift sentences in Mainland Scandinavian. In particular, Anagnostopoulou (2003, 2005) and Bobaljik (2002, 2005) note that, for varieties of Mainland Scandinavian that allow theme-recipient (Th-R) orders in passive contexts—Swedish and Norwegian—some speakers also allow Th-R orders under object shift. In Danish, which does not have theme passives, Th-R object shift constructions are also bad. We will present a more nuanced characterization of this cross-speaker and cross-linguistic variation in the following discussion, but for the moment let us summarize the relevant facts as in Table 1. Anagnostopoulou (2003, 2005) takes the facts in Table 1 to support the escape hatch movement illustrated in (3). In particular, Anagnostopoulou proposes that the same short theme movement that feeds Th-R orders in object shift also feeds theme-passivization.

**Table 1:** Theme passivization and Th-R object shift in Mainland Scandinavian

	Theme passives	Th-R object shift
Swedish/Norwegian	Yes	Some speakers
Danish	No	No

The locality approach makes a strong prediction about cross-speaker variation in Norwegian and Swedish, namely that the same speakers that accept Th-R orders in active contexts will also accept Th-R orders in passives. This paper reports on a large-sample controlled judgment experiment with speakers of Norwegian (*N* = 500) that fails to support this strong prediction of the locality approach. We propose that constraints on object ordering in these varieties are better modeled on a revised version of the case-based approach proposed by Holmberg et al. (in press). On this approach, patterns of object ordering are governed by variation in the way that phi-probes agree with theme and recipient arguments.

The situation in Scandinavian is complicated by the fact that Swedish is not an unqualified symmetrical language on a par with Norwegian. As noted by Holmberg and Platzack (1995), theme passives with verbs like ‘give’ and ‘send’ are degraded. Lundquist (2014) shows that recipient passives, too, are degraded with the verb *ge* ‘give’. However, as noted in these works, both recipient and theme passives improve when the ditransitive verb is bimorphemic, composed of a prefix plus a stem in the manner of *till-dela* ‘award’, *före-visa* ‘show’, *er-bjuda* ‘offer’. These claims are largely supported by a second experiment which we report on here. We will argue that the prefixes of the bimorphemic verbs provide an extra case-marking feature needed in Swedish.

The discussion is organized as follows. Section 2 describes the Norwegian judgment experiment and presents an analysis of the Norwegian results drawing on Haddican and Holmberg’s (2012) analysis of similar facts in British English dialects and Fox and Pesetsky’s (2005) cyclic linearization algorithm. Section 3 analyzes and discusses results from the Swedish experiment.

## Object ordering in Norwegian

### Experiment 1

We begin by describing an experiment designed to test predictions of Anagnostopoulou’s seminal proposal about symmetric passives in Mainland Scandinavian. We will focus specifically on the Norwegian forms as in (1), repeated here.

(4)

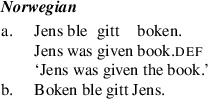

The base order for objects in Norwegian active double object constructions with full DP objects is R-Th, as shown in (5).

(5)

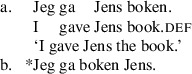

Norwegian DOCs share with their English counterparts the restriction that inanimate DPs cannot be recipients (Anagnostopoulou 2003; Green 1974; Harley 2002):

(6)

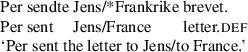

The same restriction applies in both R-Th and Th-R orders in passives, dispelling the possibility that the theme passive is derived from a covert prepositional dative.

(7)

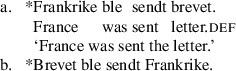

Anagnostopoulou’s (2003, 2005) proposal for Mainland Scandinavian focuses on the relationship between ditransitive passive sentences and object shift sentences with two objects, as in (8) from Norwegian.

(8)

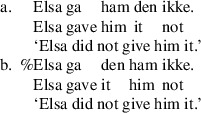

“Object shift” in the Scandinavian context refers to constructions where one or both objects raise out of the verb phrase, as diagnosed by their surface position to the left of low adverbials including the negative morpheme *ikke*. Shifting is generally restricted to weak, unstressed pronouns. In Scandinavian languages, object shift is only possible when the main verb also evacuates the VP. In perfect contexts, for example, where the participle does not raise out of VP, object shift is blocked, as shown in (9). The sensitivity of object shift to verb movement is known as “Holmberg’s generalization” (Holmberg 1986, 1999).

(9)

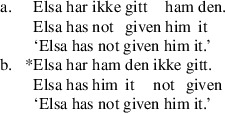

Anagnostopoulou (2003, 2005) proposed that Th-R object shift sentences like (8b) and theme passives share an abstract source, namely a derivational step moving the theme to an outer specifier of the head introducing R, which is ApplP in Anagnostopoulou’s proposal. We illustrate this in (10). In passive contexts, the higher of the two arguments will be attracted to subject position. In object shift contexts, both objects will raise into the middle field. An order preservation requirement will ensure that the lower argument “tucks in,” so that the two objects are ordered Th-R in this higher domain Richards (1997).

(10)

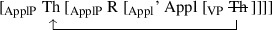

Without further assumptions, the approach in (10) should also produce Th-R orders in active contexts without object shift, such as perfect constructions where object shift is blocked by Holmberg’s Generalization. In particular, (10) should produce (11), which Anagnostopoulou reports to be categorically absent. (We will later report evidence indicating cross-speaker variation in the acceptability of such forms.) Anagnostopoulou (2003) suggests that the unavailability of (11) is due to the fact that the movement step in (10) is licit only if it feeds a subsequent movement operation, but does not provide a detailed account of this requirement.

(11)

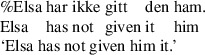

In the following discussion, we report on a judgment experiment designed to test a strong prediction made by Anagnostopoulou’s approach, namely that individual speakers will accept Th-R orders in passive contexts if and only if they also accept Th-R orders in OS. Participants in the experiment were 500 self-described native speakers of Norwegian, recruited online by the researchers. Subjects ranged in age from 18 to 81 (*M* = 38.9, *SD* = 11.5); 371 were women. Participants were not compensated for their participation.

The experiment crossed two factors: *object order*, with levels R-Th and Th-R, and *context*, with three levels, passives, active OS and active in situ. Examples of these six conditions are given in Table 2. All theme and recipient arguments were third person pronouns. Theme vs. recipient interpretation of arguments was biased using animate pronouns for recipients and inanimate pronouns for themes. We used the Bokmål standard for all stimuli and instructions to participants. A list of the experimental items appears in Appendix 1.

**Table 2:** Examples of six experimental conditions

Context	Theme-Recipient	Recipient-Theme
**Passives**	Den ble gitt ham.	Han ble gitt den.
	‘It was given him.’	‘He was given it.’
**Act. OS**	Elsa ga den ham ikke.	Elsa ga ham den ikke.
	‘Elsa didn’t give him it.’	‘Elsa didn’t give him it.’
**Act. In situ**	Elsa har ikke gitt den ham.	Elsa har ikke gitt ham den.
	‘Elsa hasn’t given him it.’	‘Elsa hasn’t given him it.’

12 lexicalizations were created for each of these six conditions, which were then blocked and assigned to one of 12 lists by Latin square. Each list contained four items per condition for a total of 24 critical items. (Each subject saw each lexicalization twice.) These 24 sentences were pseudo-randomized with 24 fillers, half of which were grammatical and half ungrammatical. Subjects were pseudo-randomly assigned to lists by a counter mechanism in the experimental application, Ibex (Drummond 2013).

The experiment was self-paced, through a web-based application. The application displayed sentences one-by-one accompanied by an array of square icons numbered zero through ten in order, horizontally, from left to right. The endpoints of the scale were labeled *dårlig*, ‘bad’ and *god*, ‘good’, respectively. Participants entered their rating for each sentence by typing a number from their keypads or clicking on one of the eleven icons. The application did not permit subjects to view previously judged items or to change previously given ratings.

Table 3 summarizes the fixed effects from a linear mixed effects regression models with random intercepts for subject and lexicalization and a by-subject random slope for order. The model was fit using the lme4 package in R, with reference levels Order = Recipient-Theme and Context = In situ (R Core Team 2014; Bates et al. 2015).^1^

**Table 3:** Fixed effects of a model of acceptability scores

	*β*	*t*	p
(Intercept)	7.149	27.32	<.00001
Order = Th-R	−5.976	−60.86	<.00001
Context = Active-OS	−1.115	−14.28	<.00001
Context = Passive	−1.228	−15.72	<.00001
Order = Th-R:Context = Active-OS	1.560	14.12	<.00001
Order = Th-R:Context = Passive	7.020	63.52	<.00001

**Fig. 1:**
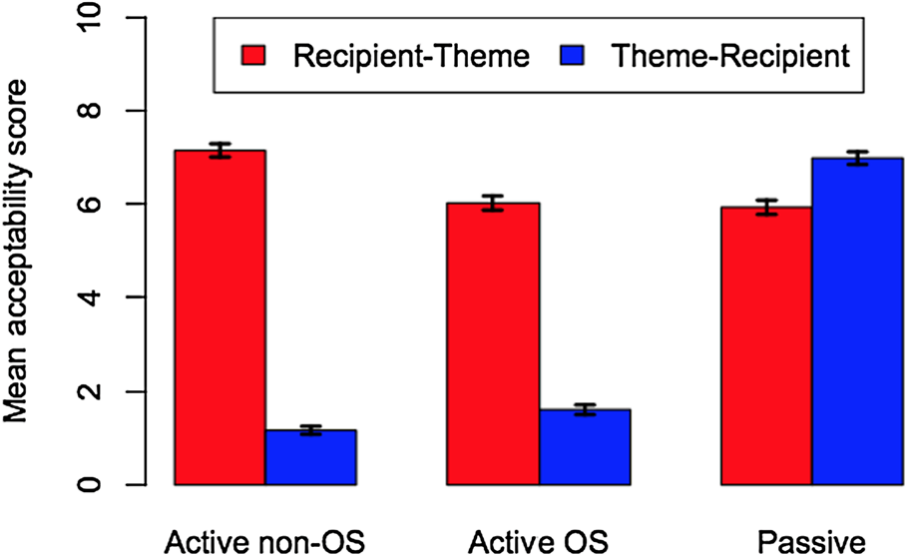
Tendency toward theme-recipient order in active contexts

The analysis revealed a significant interaction between context and object order, as illustrated in Fig. 1, which shows mean scores and 95% confidence intervals for the six conditions. Figure 1 shows that the two passive conditions are judged fairly positively, with scores for theme-passives somewhat better than those for recipient-passives. For the active conditions, participants on aggregate accepted R-Th orders but gave fairly low ratings to Th-R sentences.^2^

**Fig. 2:**
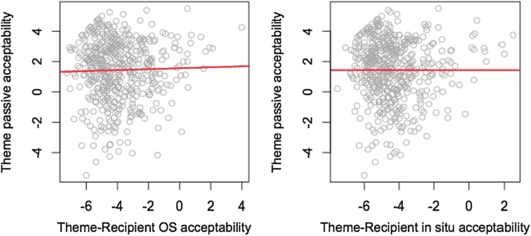
By speaker contrasts in actives and passives

The principal measures of interest from the perspective of the locality approach are whether acceptance of Th-R orders in the two active conditions correlates across participants with acceptance of Th-R orders in passives. We illustrate these relationships in Fig. 2, which plots by-speaker contrasts between Th-R orders in these three conditions and the filler scores; that is, for each axis, (mean score for Th-R order)-(mean score for fillers). (Because half of the fillers were grammatical and half ungrammatical, zero on each axis might be taken as a crude mid-point of acceptability.) The two plots show a negligible relationship in both cases (*r* = .03, *p* = .540 for the object shift condition, *r* = −7.10e–05, *p* = .9987 for the active *in situ* condition). The results in Fig. 2, therefore, provide no support for the locality approach illustrated in (3).

**Fig. 3:**
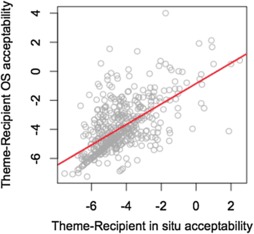
By speaker contrasts in active object shift and unshifted contexts

A third possible relationship to consider is the relationship between the two active conditions. Figure 3 plots the same by-speaker contrasts for active object shift and active non-object shift sentences. The figure shows a positive, highly significant relationship (*r* = .620, *p*<.00001), between these conditions, indicating that speakers generally accept Th-R orders vP-internally to the same, typically low, degree as they do under object shift. We propose a model of these results in the next section.

### Modeling Th-R orders: Agreement and order preservation

The results presented above pose two main problems for formal theories of ditransitives. A first is to explain the fact that acceptance of Th-R orders in object shift and active non-object shift contexts correlates across speakers. A second is to model the cross-speaker variation in acceptance of Th-R orders in active and passive contexts. Abstracting away from the gradience in the responses for a moment, we note that Fig. 2 shows that all four logically possible patterns of responses in acceptance of Th-R orders in active and passive contexts are instantiated: some speakers accept Th-R orders in both active and passive contexts, some in neither, and some only in one of these contexts. We take these four possibilities to reflect four relevant grammars, and we summarize these four possibilities in Table 3 (as evident from Fig. 2, only a tiny minority of speakers have Grammars 2 and 3, though). We take gradience in judgments to reflect grammar competition in the sense of Kroch (1989). Probabilities over competing representations, which vary from subject to subject, will determine the cross-speaker variability illustrated in the plots above.

Following much other recent work on the DOC, we assume that there is an abstract head involved in the DOC. The exact structural function and position of this head is subject to much controversy; see McGinnis (2001), Harley (2002), Pylkkänen (2008), Anagnostopoulou (2003), Baker and Collins (2006), Bruening (2010a,b), Harley and Jung (2015). We will here follow Harley and Jung (2015), building on Harley (2002, 2008), and assume the following structure for the vP of *John gave Mary a book*.

(12)

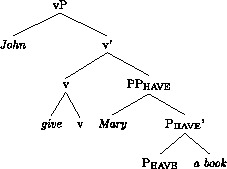

The general idea, going back to Kayne (1984), is that the two objects form a small clause. A more recent development of this hypothesis is that *give* and other verbs in this class (*send, pass, hand*, etc.) mean ‘cause to have’, where v in (12) provides the ‘cause’-element (Harley 1996, 2002; Beck and Johnson 2004; see Harley and Jung 2015 for a recent defence of this analysis against critiques in Pylkkänen 2008 and Bruening 2010a). Under this hypothesis, v assigns the Agent theta-role of the subject, while P_have_ assigns the theta-roles of both objects. An unusual feature of the theory underpinning this analysis is that the verb itself is merged directly with v. Harley and Jung, following Harley (2002), refer to this as “manner adjunction”; the lexical verb specifies the manner in which the recipient (R) gains possession of the theme (Th).

An alternative approach developed since the early 2000s is that the DOC involves an abstract “applicative” head, which assigns a theta role to one of the arguments, or both, depending on theoretical assumptions (McGinnis 2001; Pylkkänen 2008; Bruening 2010a). The generalizations that we will discuss, based on findings from experimental research that we will report, do not, for the most part, crucially depend on the choice between these approaches.

We assume, quite uncontroversially, that one of the objects in a DOC is assigned the case that transitive v assigns to a DP. The question then is, where the other, “extra” case comes from. We propose that one locus of cross-speaker (and cross-linguistic) variation in the distribution of Th-R vs. R-Th orders is in the way that P_have_ assigns case. In particular, let us assume two distinct representations in competition in the Krochian sense (Kroch 1989, 1994, 2001). One variant might be termed the “standard model” whereby phi probes agree downwards with the closest target with matching active features. In active contexts, v will agree with the recipient argument, and P_have_ will agree with the theme. In passive contexts, in which v does not introduce an external argument and is not a source of case, the recipient will move to TP, where it receives nominative case. We illustrate this scenario in (13), the dashed arrows here, and in subsequent trees, representing case-assignment.^3^

(13)

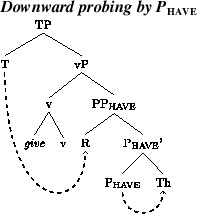

A second, competing representation, following Holmberg et al. (in press), is one where P_have_ agrees with and assigns case to the recipient argument in its spec. The recipient, being assigned case independently and being thereby deactivated (Chomsky 2001), does not intervene for subsequent case assignment to the theme. In active contexts, the theme will be probed by v. In passives, v, again, will not be a probe and the Theme will be probed by T and attracted to its specifier. On this approach, therefore, theme-passives are made possible by agreement between P_have_ and the recipient argument in its specifier, together with the assumption that the inactive recipient does not intervene for subsequent agreement operations with the Theme. We illustrate this proposal in (14). We propose that gradience in intuitions of well-formedness of these forms reflects competition between this grammar and the downward-probing option described in (13).^4^ (To express the four-grammar pattern in Table 4, we will shortly posit an additional locus of variation in active contexts.)

**Table 4:** Availability of Th-R orders in active and passive contexts

Grammar	Th-R actives	Theme passives
1	*	*
2	Ok	Ok
3	Ok	*
4	*	Ok

(14)

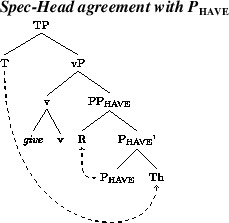

Assignment of case to the recipient by the head of the DOC is familiar from languages that have overt, dative case on the recipient, like German and Icelandic. In these languages, we take it that the dative is inherent case, assigned along with the theta role and preserved under movement (Holmberg and Platzack 1995: Ch. 7). In German, it has the consequence that the recipient cannot be passivized, as the subject of the passive can only be nominative (McFadden 2004; Georgala 2011a,b). In Icelandic, where dative DPs can be subjects, the recipient can be passivized, preserving its dative case (Holmberg and Platzack 1995: Ch. 7). The case-assignment in Norwegian (14) may be vestigial inherent case. There are dialects in Norwegian which have preserved (some) dative case, including, for some of them, on the recipient in the DOC (Eyþórsson et al. 2012). While this is a strong indication that the configuration (14) obtains in those dialects of Norwegian, we cannot draw any interesting conclusions from it for other dialects.^5^ In most varieties of Norwegian, the only evidence of (14) in the primary linguistic data is the existence of theme passives. We take this to be sufficient evidence, though, for language learners to adopt (14). On the other hand, the existence of recipient passives in the primary linguistic data is evidence that the case configuration in (13) is an option as well.^6^

We assume, in keeping with current phase theory, that in passive contexts, objects do not raise to TP in a single movement step but rather stop off in spec, vP, which has an EPP feature. This assumption, however, has no substantive consequences for the analysis.

Let us now consider how these assumptions help in modeling the Norwegian results above. Consider, first, the derivation of the recipient passive in (1a), repeated here.

(15)

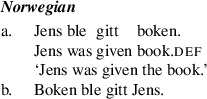

Such sentences will be generated when P_have_ agrees downward with the theme. The recipient will subsequently raise to spec, vP, where it will be probed by T. We illustrate this in (16).

(16)

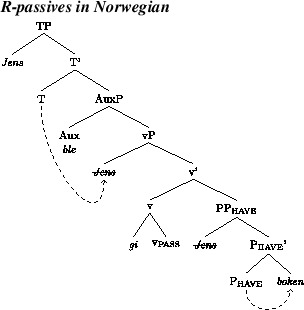

Consider, next, theme passive sentences like (15b). Such forms will be produced when P_have_ agrees with the recipient argument that it introduces. This allows passive v to probe the theme. Given that passive v has an EPP-feature, this will trigger movement of the theme to spec of vP, as indicated in (17). This, in turn, allows T to probe the theme, assign case to it, and trigger movement of it to spec of TP.

(17)

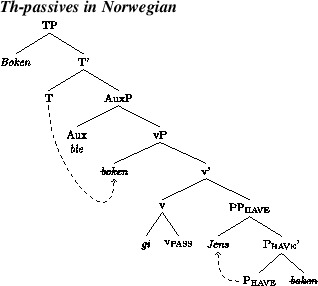

Additional assumptions will be needed to explain the word order variation in active contexts. The fact that the acceptance of Th-R orders in passive contexts does not correlate across speakers with acceptance of Th-R orders in actives suggests that the latter involves an additional locus of variation. In addition, the fact that relative acceptance of Th-R orders under object shift and vP-internally correlate positively across speakers (see Fig. 3) suggests a single locus of variation governing word order in these two contexts. We propose that the locus of variation is whether the theme can move to the edge of P_have_, a property which can be formally represented as an EPP feature on P_have_. More specifically, we posit two representations in competition. One will lack an EPP feature on P_have_, leaving both arguments *in situ* and resulting in a Th-R linear order. A second representation will involve an EPP feature on P_have_, which will attract the theme to its spec, as in (18), producing recipient-theme orders in active contexts. This would be a movement step that does not affect case relations, a form of scrambling. The theme would still be assigned its case by P_have_, as normal in the active DOC, and the recipient would be assigned its case by v.

(18)

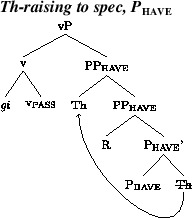

Something more, however, is needed to explain why Th-R order is preserved in object shift contexts. One possibility is that order preservation under object shift is a consequence of the fact that the object shift movement operation targets a constituent containing both objects. A principal disadvantage for such an approach is that extra-VP adverbials like *sjølsagt*, ‘obviously’ can intervene between the two objects in shifted contexts. This indicates that OS can move objects separately.

(19) Jeg ga ham sjølsagt den ikke.
  I gave him obviously it not
  ‘I obviously didn’t give him it.’

We pursue an alternative approach based on the observation that the object order preservation effect described above applies in exactly the same environments—object shift contexts—that a better studied shape preservation effect applies, namely Holmberg’s Generalization. Our analysis of the cross-speaker correlation in object-order preference in the two active contexts will be based on a theory of linearization originally motivated in part by Holmberg’s Generalization effects, namely Fox and Pesetsky’s (2005) Cyclic Linearization proposal. There are several formal approaches to order preservation effects (Sells 2001; Richards 2004; Fox and Pesetsky 2005; Engels and Vikner 2013). Here, we use Fox and Pesetsky’s system, which expresses the relevant facts without further assumptions.^7^

Fox and Pesetsky propose that precedence relations among syntactic objects are established phase-by-phase. When a given constituent is spelled out, terminal elements are mapped to precedence relations, which are later linearized in the phonological component. Any movement operations applying to a syntactic object already spelled out must preserve the relative order of terminal elements in the previous phase, since, otherwise, spell out of the higher phase would produce a precedence relation that conflicts with one established in the lower phase. The conflicting precedence relations would then not be linearizable. Consider, for example, the case of two objects, X and Y, merged inside a phase, Phase 1. Spell out will establish the order <X,Y>. (Fox and Pesetsky call such ordered pairs, interpretable by the phonology, *ordering statements*.)

(20) [Phase1 X, Y ] → <X,Y>

Now, consider a case where Y, but not X, moves into a higher phase, establishing the order <Y,X> at the point of spell out of the higher phase. If Y moves in one fell swoop, as in (21a), the sets of precedence relations established in the two phases will conflict—<X,Y> established by the first application of spell out and <Y,X> by the second—and the result will not be linearizable. If, however, Y moves within Phase 1 to a position preceding X the precedence relations established will be <Y,X> for both phases, and no conflict arises. Fox and Pesetsky’s approach, therefore, derives successive cyclicity from a theory of order preservation.

(21)

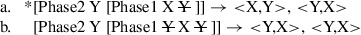

For our purposes, what will be important is a context in which multiple objects remerge in a higher phase. If extra-phasal movement permutes the relative order of the two elements, spell out of the higher phase will necessarily fix a precedence relation different from that established in the lower phase, as shown in (22a). If, on the other hand, movement preserves the relative order of these two elements as in (22b), the precedence relations established in the higher phase will match those in the lower phase, and no conflict arises.

(22)

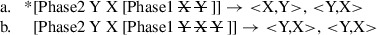

Let us now apply this framework to order preservation effects under object shift. As Holmberg (1999) observes, word orders in embedded clauses suggest that the landing site of Scandinavian object shift is a position in the middle field, above vP. In (non-root) embeddings where V-to-C movement does not apply, the negative morpheme, *ikke* appears to the left of the perfect auxiliary, as shown in (23).

(23)

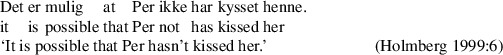

If we take sentences like (23) to faithfully reflect the first-merged orders of *ikke* and the auxiliary, then it must be the case that the first merged position of *ikke* is above the first merged position of auxiliaries, given the right branching structure of the lower clause in (23). We therefore take object shift to target a position above negation as in (24). For our purposes, it will not matter what exactly this landing site is, and we refer to it here as “FP.”

(24) [tp T [FP Obj F …*ikke* … [vp V Obj ] ] ]

Given that object shift targets a position across a phase boundary from the first merged position of the objects, cyclic linearization immediately expresses the object order preservation effects under object shift. That is, permutation of the relative order of the objects under object shift is possible if and only if the objects permute in a lower phase. As mentioned, we propose that this is movement to an outer specifier of PP_have_, as shown in (18). When the vP phase is spelled out/linearized, the linear order between the arguments will be Th-R. This order will be preserved under object shift, under the theory of linearization in Fox and Pesetsky (2005).

On the face of it, the movement in (18) is just the kind of short theme movement that Anagnostopoulou (2003) envisaged. However, what our results show is that there is no relation between this short movement and theme passives in Norwegian. The short theme movement is very much a minority phenomenon while theme passives are ubiquitous.

### British English dialects

Somewhat similar patterns of cross-speaker variation have recently been described for British English dialects (Biggs 2014, 2015; Haddican 2010; Haddican and Holmberg 2012; Myler 2011, 2013). In this section, we consider how the framework just introduced might be extended to object order variation in these varieties.

Much previous literature has noted that some speakers of British English dialects accept theme passives in addition to recipient passives (Anagnostopoulou 2003; Doggett 2004; Woolford 1993) as in (25). Less discussed in the formal literature until fairly recently is the fact that many Northern and Western British English dialects allow for Th-R orders in active double object sentences in addition to R-Th orders as in (26). The co-occurrence of (25b) and (26b) in these dialects suggests the possibility of a single abstract source—a short theme-movement operation as in (3).^8^

(25)

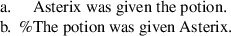

(26)

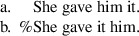

The predictions of the locality approach for these facts are similar to those outlined above for Norwegian: speakers should accept Th-R orders in passives if and only if they also accept Th-R orders in the active context in (26b). (Haddican and Holmberg note that the objects in (26) are unlikely to have undergone object shift—at least not as high as that in Norwegian—and so these sentences are comparable to the active non-object shift sentences in Table 2.) To test this prediction, Haddican and Holmberg carried out a judgment experiment with 137 native speakers of Northern and Western British English dialects. Their results revealed a positive correlation across speakers in acceptance of (25b) and (26b) as indeed expected from the perspective of the locality approach. Nevertheless, Haddican and Holmberg noted two main facts about these dialects that are problematic from the perspective of the locality hypothesis. A first concerns cross-speaker variation in the data. Some speakers in the sample accepted Th-R orders in both passive and active contexts, and others accepted neither, which is expected on the locality approach. In addition, however, many speakers in these dialect areas accept Th-R orders in active contexts but not in passive contexts. This is not predicted by the locality approach, since the availability of movement to the “escape hatch” position should necessarily make available theme passivization. No speakers in Haddican and Holmberg’s sample showed the fourth possible pattern: acceptance of theme-passives but not Th-R orders in actives. These patterns of responses correspond to Grammars 1, 2 and 4 in Table 4 for Norwegian. The British English counterpart of Norwegian Grammar 3 is unattested in Haddican and Holmberg’s sample.

A second obstacle for a locality approach to (25) and (26) has to do with a restriction on themes in active but not passive contexts. The theme in Th-R active sentences in the relevant dialects must be a weak pronoun. Compare (26b) and (27).

(27) *She gave the book him/John.

No such restriction on themes applies in passives; that is, both pronominal and full DP themes are possible, as in (25b). From the perspective of the locality approach illustrated in (3), these facts seem to require that full DPs can move through the escape hatch position, but only if they move on subsequently to subject position (or a phase edge).

The speakers that accept the Th-R order in the active or the passive have a grammar in which R is assigned the “extra case” of the DOC allowing Th to be assigned the “regular” objective case by v. Haddican and Holmberg (2012) propose, in part following Baker and Collins (2006), that these grammars have an additional head, a Linker, which assigns case to R. For the sake of exposition, we will reformulate Haddican and Holmberg’s analysis in present terms; this will not affect the principal claims made in that paper. The structure of the DOC is (12), repeated here.

(28)

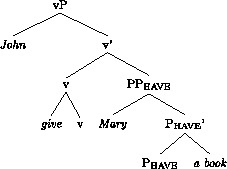

In the grammars responsible for the Th-R order, P_have_ assigns case to R, deactivating it, which allows v to probe Th across R. Following Chomsky (2001), the Agree relation between v and Th entails that v’s unvalued set of phi-features are valued by copying the phi-feature values of the object, while the object is assigned objective case. In the case where Th is a weak pronoun, taken to be a pronoun with no D but phi-features only (following Cardinaletti and Starke 1999), the features of the pronoun will thereby be a subset of the features of v. Following Roberts (2010), this means that v and the pronoun form a chain of two copies. By the ordinary chain reduction rule at PF (Nunes 2004), the higher copy will be spelled out, the lower copy will not. Roberts (2010) refers to this as “incorporation” of the goal into the probe. Now, the English pronoun *it* is a weak pronoun. Following incorporation, the order in an active DOC with *it* as Th will be as in (26b), with *it* spelled out preceding R. In contexts with full DP themes, no such incorporation will apply and the theme will spell out in its lower position. The structure in (28), together with Roberts’ (2010) incorporation proposal therefore gives the desired result that, in this grammar, inversion is only possible with weak themes.

In passive contexts, where no external argument is merged, Haddican and Holmberg assume, as is standard, that passive v is not a case assigner. In the structure in (28), now taken to be a passive and therefore without an external argument, P_have_ will assign case to R. In a subset of these grammars, passive v has an EPP feature, triggering movement of Th to the edge of vP, where it can then be probed by T, be assigned nominative case, and move on to spec,TP (active transitive v always has an EPP feature checked by the external argument). The grammar with Th-R order in active but not passive contexts differs minimally from the above in that v lacks an EPP feature triggering movement of Th to the spec of vP. Given that a direct Agree relation between T and the theme in vP is impossible, because either passive vP or P_have_/Appl is a phase (in the sense of Chomsky 2000, 2008), the theme is thereby trapped case-less in situ.

Haddican and Holmberg’s (2012) results from Northwest British English dialects provide additional support for the role of case vis-à-vis locality in modeling passive symmetry. Their analysis, moreover, is close in spirit to the proposals for Norwegian presented above. What crucially distinguishes NW British English from Norwegian is the fact that the latter has an additional operation, Object Shift, absent in the former. (See Haddican and Holmberg 2012 and Holmberg 1999 for discussion.) We turn to a final set of data supporting a case-based approach to passive symmetry in the next section.

## Verb class effects in Swedish

In the remaining discussion, we focus on passivization out of double object constructions in Swedish. As in English and Norwegian, the base order of double object constructions is R-Th (Holmberg and Platzack 1995: 190–194). Double object constructions in the order Th-R orders with two full DPs are sharply degraded as shown in (29b).

(29)

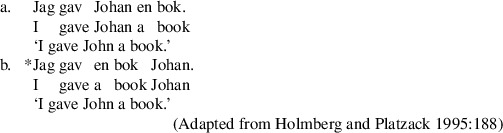

As in Norwegian and English, recipient arguments in Swedish DOCs must not be inanimate as in (30).

(30)

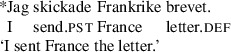

We therefore take Swedish sentences of the kind in (29a) to be bona fide DOCs, as standardly assumed (Holmberg and Platzack 1995: 190–199). We assume, therefore, that the structure of the Swedish DOC is like the English structure (12), (repeated here), as assumed previously in the analysis of Norwegian.

(31)

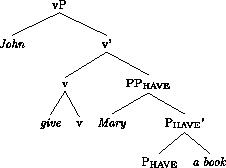

In the A-movement literature, Swedish is sometimes described as a symmetric passive language (Woolford 1993; Anagnostopoulou 2003; Platzack 2005). As we will show, this is indeed the case, but not in the way it is usually portrayed. First, as noted by Holmberg and Platzack (1995), with many garden variety ditransitive verbs including *ge*, ‘give’, theme passives are quite poor.

(32)

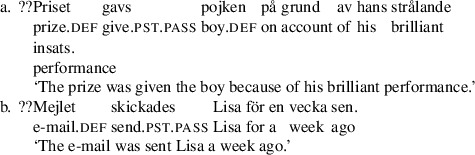

This effect was described by Lundquist (2004) based on corpus results. However, his investigation did not show any significant difference between R-passives and Th-passives of the verb *ge* ‘give’; both were about equally rare. This was further confirmed in a dialect survey reported in Lundquist (2014): out of 192 speakers in 48 locations distributed all over Sweden and the Swedish-speaking parts of Finland (4 speakers per location) close to 92% rejected the sentence (we represent this as ??).^9^

(33)

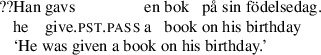

However, Holmberg and Platzack (1995:219–220) report that theme passives are better with a class of bimorphemic verbs, including *tilldela*, ‘award’, *tillskriva* ‘ascribe’, and *förära* ‘award’, as in (34):

(34)

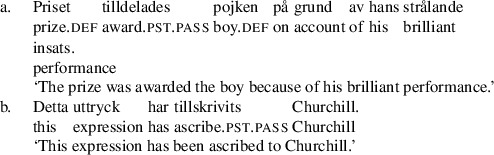

The first morphemes in some of these verbs are homophonous with prepositions, including *till* ‘to’, an observation which was taken to be significant by Holmberg and Platzack (1995). The generalization was confirmed by Lundquist’s (2004) investigation. But, again, his investigation showed the same effect for R-passives, which also improve with this class of verbs.

(35)

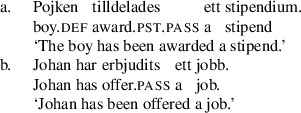

From the perspective of the approach to passive symmetry proposed in the previous section, one possible understanding of the contrast observed by Holmberg and Platzack (1995) and Lundquist (2004, 2014) between (32) and (33) on the one hand, and (34) and (35) on the other hand, is (a) the passive formed with a simple ditransitive verb such as *ge* ‘give’ fails to assign case to one of the objects which remains in vP, and (b) the preposition-like prefixes provide the extra source of case otherwise absent.

A possibility which should be controlled for is that the bimorphemic verbs in (34) and (35) are derived from the V+PP construction rather than the DOC. This can be tested with the verb *tillsända* ‘send’.

(36)

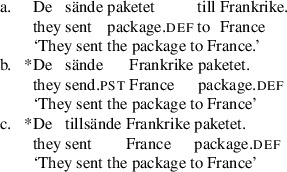

The bimorphemic alternative here patterns with the DOC, not with the V+PP construction, as it needs an animate receiver, which follows if the constructions in (34) and (35) are passives of the DOC under the ‘cause to have’ hypothesis that we have adopted here.

Many Swedish speakers, including linguists that we have consulted, find the contrasts in (32)–(35) quite subtle, and judgments vary from speaker to speaker. To understand these facts better and to examine possible between-subjects effects, we carried out a controlled judgment experiment. Subjects in the experiment were 101 self-described native speakers of Swedish aged 22 to 87 (*M* = 38.4, *SD* = 12.0); 66 were women. Subjects were recruited online by the researchers. We did not require subjects to be linguistically naïve. Subjects were not compensated for their participation.

The experiment crossed two factors: *object order*, with levels R-Th Th-R, and *verb class*, with two levels: monomorphemic and bimorphemic. All theme and recipient arguments were third person pronouns. Theme vs. recipient interpretation of arguments was biased using animate pronouns for recipients and inanimate pronouns for themes. We summarize these four conditions in Table 5.

**Table 5:** Example sentences for 4 conditions

Context	Theme-passive	Recipient-passive
**Monomorphemic**	Priset gavs pojken.	Pojken gavs priset.
	‘The prize was given the boy.’	‘The boy was given the prize.’
**Bimorphemic**	Priset tilldelades pojken.	Pojken tilldelades priset.
	‘The prize was awarded the boy.’	‘The boy was awarded the prize.’

The procedure for the experiment was similar to that of the Norwegian experiment described above. Twelve lexicalizations were created for each of these four conditions. These were blocked and assigned to one of twelve lists by Latin square. Each subject saw four items per condition. These 16 sentences were pseudorandomized with 24 filler sentences: half grammatical, half ungrammatical. Subjects judged each of these sentences on an 11-point scale with endpoints labeled *dålig* (‘bad’) and *bra* (‘good’). Subjects were assigned to lists by the software using a counter. A list of the experimental items appears in Appendix 2.^10^

A possible confound in the experiment concerns stylistic effects. If theme passives are associated with more formal speech events, then any verb class-effect could conceivably be attributable to a register effect rather than a grammatical one. To address this possibility, the acceptability judgment task described above was followed by a formality judgment task. Participants were asked to judge the formality of theme-passive vs. recipient-passive sentences taken from the monomorphemic condition. Subjects judged four sentences from both of these two conditions along with eight fillers on an eleven-point (0–10) scale, with endpoints labeled *informella* (‘informal’) and *formella* (‘formal’), respectively.

Table 6 summarizes the fixed effects from a linear regression model with random intercepts for subject and lexicalization and a by-subject random slope for order. The reference levels for the morphological factor was Bimorphemic and for the order factor Recipient-Theme.^11^

**Table 6:** Fixed effects of a model of acceptability scores from Swedish experiment

	*β*	*t*	p
(Intercept)	6.3867	15.881	4.02e−12
Morphology = Monomorphic	−1.6531	−8.480	< 2e−16
Order = Th-R	−0.4307	−2.162	0.03137
Morphology = Monomorphic:Order = Th-R	−0.7501	−2.728	0.00645

**Fig. 4:**
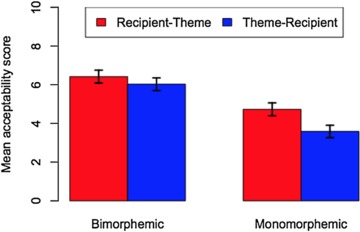
Mean scores and 95% CIs for four conditions

The analysis revealed a significant interaction between verb class and object order, as illustrated in Fig. 4, which shows mean scores and 95% confidence intervals for the four conditions. Figure 4 shows that both passive types are rated fairly low with monomorphemic verbs, as originally noted by Lundquist (2004). Both passive types improve in the bimorphemic condition. In addition, Fig. 4 illustrates an interaction between verb class and word order: theme passives are judged roughly on a par with recipient-passives with bimorphemic verbs, but much lower in the monomorphemic condition. The fact that theme-passives improve relative to recipient-passives with bimorphemic class verbs is in line with Holmberg and Platzack’s (1995: 219–220) observation. We return to this effect shortly.^12^

An analysis of the results from the second subdesign focusing on a possible stylistic difference between theme-passives and recipient-passives revealed no effect. To test further for possible stylistic effects, by-subject random slopes from a model of the subdesign 2 results were included as covariates in the modeling of the verb class*order interaction. This factor did not contribute significantly to the model.^13^

We take the fact that both R-passives and Th-passives are degraded with monomorphemic verbs to suggest that v is involved in the licensing of both arguments in active contexts. Given standard assumptions about case and the passive this conclusion is almost inescapable. We propose that Swedish differs from Norwegian in that, in active contexts, there is “multiple agreement”—that is, a single probe that values case on both theme and goal arguments (Anagnostopoulou 2003; Nevins 2007, 2011). We take this probe to be v, as illustrated in (37). In passive contexts, we propose that this head fails to assign case, as in Norwegian, with the consequence that, in passive contexts, one of the two internal arguments will necessarily be left case-less in violation of the case filter. These assumptions together, therefore, express the fact that passivization of both arguments out of Swedish DOCs is degraded. In addition, to the extent that passives are available with this class of verbs they are accepted with R passives. We take this to mean that the grammar where P_have_ is a source of case is marginally available. The fact that Th-passives are sharply degraded with monomorphemic verbs suggests that, unlike in Norwegian, the grammar where P_have_ agrees with its specifier is unavailable in Swedish.

(37)

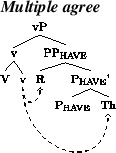

We propose that, in the case of Swedish bimorphemic verbs, the prefixal P provides an extra source of case. Specifically, let us assume that Swedish bimorphemic verbs of the kind described above are complex heads with the structure in (38).

(38) [_v_ [_v_ P V ] v ]

Let us further assume that P’s properties as a probe—that is, its uninterpretable *ϕ* features—transfer to v, but only in the case of passive v, where v itself lacks uninterpretable *ϕ* features. Enriched with the *ϕ* features of P, v probes and agrees with the recipient—the closest active goal—with the consequence that the theme will be case-less until subsequently probed by T. We illustrate this variation in (39). The assumption of an additional source of case outside of the projection containing the two objects, together with the assumption that inactive recipients need not defectively intervene, immediately expresses the fact that bimorphemic verbs selectively ameliorate theme passives.^14^ In addition, Fig. 4 shows that R-passives also improve to some degree with bimorphemic verbs, though to a lesser extent than Th-passives. Following the logic of our argument so far, we suggest that the grammar just proposed may compete with a minority variant where the *uϕ*-features transmitted from P to v are transmitted once more to P_have_, and thus probe the theme, leaving the recipient to be probed by T.

(39)

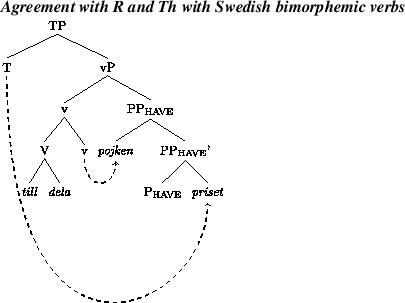

Under this theory, passive symmetry in Norwegian and Swedish have different sources, both related to mechanisms of case assignment. In Norwegian, Th-passives are made possible by a grammar in which P_have_ may assign case to the recipient in its specifier, enabling promotion of the theme. In Swedish, the fact that both R-passives and Th-passives are degraded with monomorphemic, *give*-class verbs, suggests that P_have_ can only marginally be a case-assigner in Swedish, which is “symmetric” with this class of verbs only in the sense that neither passive is good. What improves Th-passives in Swedish is the presence of an additional source of case above PP_have_, namely the prepositional prefix.^15^

A final point to consider is whether there is any explanation for why Swedish should differ from cognate languages like Norwegian, Danish, and English in the way it disallows or disprefers passives of simple ditransitive verbs. Another striking difference between Swedish and the other languages is that Swedish has a synthetic passive as the unmarked passive form where the other languages have a periphrastic passive as the unmarked form (Lundquist 2009; Laanemets 2013). The synthetic passive is formed with an affix *–s* on the verb (generally regarded as historically derived from the 3rd person reflexive pronoun). Could there be a connection between having the *s*-passive as the unmarked form and disallowing passives of the DOC? We can test this, because Norwegian (as well as Danish) also employs the *s*-passive, although only in the present tense and in construction with a modal verb. The question, then, is how Norwegian *s*-passives, as in (40) compare with the periphrastic passives, as in (41).

(40)

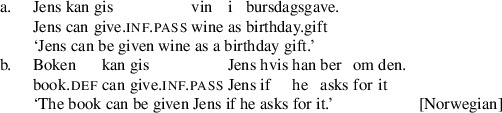

(41)

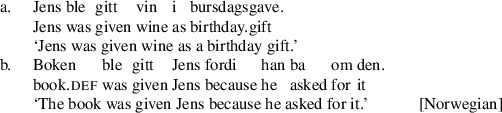

According to our Norwegian informants there is no difference between (41) and (40); they are equally acceptable. We see no reason, therefore, to think that the choice between the periphrastic and the synthetic passive makes any difference in the DOC.^16^

## Conclusion

This paper has described two large-sample judgment experiments with speakers of Swedish and Norwegian that have three main outcomes relevant to current formal approaches to the passive symmetry problem. First, the Norwegian results fail to support a prediction of Anagnostopoulou’s (2003) seminal locality-based approach to passive symmetry in Norwegian, namely that speakers should accept theme-passives if and only if they accept Th-R orders in object shift. Second, results from a survey of Swedish speakers support observations by Holmberg and Platzack (1995) and Lundquist (2004, 2014) of a verb class effect on passivization out of double object constructions: both theme- and recipient passives are degraded with *give*-class verbs, and both ameliorated with a set of verbs with preposition-like prefixes. This effect is not predicted by the locality approach. Third and finally, the Norwegian results suggest a shape conservation effect in object shift contexts not previously reported in the literature. Th-R orders in Norwegian object shift contexts are available for just those speakers who also accept Th-R orders in active non-object shift contexts. This object ordering constraint applies in the same environment that another, much better described ordering constraint applies, namely Holmberg’s Generalization. We have shown that these results are explained by Fox and Pesetsky’s (2005) cyclic linearization proposal together with the assumption that Th-R orders vP-internally reflect short theme-movement within the vP phase.

We have assumed the theory of the DOC advocated by Harley (2002, 2008) and Harley and Jung (2015) whereby the DOC means ‘cause to have’ and the two objects form a small clause headed by a head P_have_. There is variation among languages with a DOC regarding how case is assigned to the objects. In one type, the recipient is assigned the regular object case by v, in the active voice, and nominative by T in the passive, while the theme is assigned ‘extraordinary case’ by P_have_. The result is that R-passives are well formed, but Th-passives cannot be derived. Standard English and Danish would be representatives of this type. In another type, the recipient is assigned extraordinary case by P_have_ in a spec-head configuration. The theme can then be assigned the regular object case by v. This would be the case in languages with a DOC where Th-passives are well formed but R-passives are not. German would be a representative of this type. In a third type of language, represented by Norwegian, both grammatical options are available, and as a result R-passives and Th-passives are both well-formed. In yet another type of language, represented by Swedish, the objects are both assigned case by v in the active voice, with the result that neither passive is well-formed, since one of the objects will always be left without a case. Swedish has a class of verbs, though, with a prepositional prefix, which can provide the extraordinary case needed to derive Th-passives and R-passives.

## Appendix 1: Tables 7 and 8

**Table 7:** Experimental items for Norwegian survey

Order	Context	Norwegian	English
Th-R	Passive	Den ble gitt ham.	It was given him.
Th-R	Active-OS	Elsa ga den ham ikke.	Elsa didn’t give it him.
Th-R	Active-non-OS	Elsa har ikke gitt den ham.	Elsa hasn’t given it him.
R-Th	Passive	Han ble gitt den.	He was given it.
R-Th	Active-OS	Elsa ga ham den ikke.	Elsa didn’t gave him it.
R-Th	Active-non-OS	Elsa har ikke gitt ham den.	Elsa hasn’t given him it.
Th-R	Passive	Den ble vist ham.	It was showed him.
Th-R	Active-OS	Jon viste den ham ikke.	Jon didn’t show it him.
Th-R	Active-non-OS	Jon har ikke vist den ham.	Jon hasn’t showed it him.
R-Th	Passive	Han ble vist den.	He was showed it.
R-Th	Active-OS	Jon viste ham den ikke.	Jon didn’t show him it.
R-Th	Active-non-OS	Jon har ikke vist ham den.	Jon hasn’t showed him it.
Th-R	Passive	Den ble solgt ham.	It was sold him.
Th-R	Active-OS	Peter solgte den ham ikke.	Peter didn’t sell it him.
Th-R	Active-non-OS	Peter har ikke solgt den ham.	Peter hasn’t sold it him.
R-Th	Passive	Han ble solgt den.	He was sold it.
R-Th	Active-OS	Peter solgte ham den ikke.	Peter didn’t sell him it.
R-Th	Active-non-OS	Peter har ikke solgt ham den.	Peter hasn’t sold him it.
Th-R	Passive	Den ble sendt ham.	It was sent him.
Th-R	Active-OS	Jens sendte den ham ikke.	Jens didn’t send it him.
Th-R	Active-non-OS	Jens har ikke sendt den ham.	Jens hasn’t sent it him.
R-Th	Passive	Han ble sendt den.	He was sent it.
R-Th	Active-OS	Jens sendte ham den ikke.	Jens didn’t him it.
R-Th	Active-non-OS	Jens har ikke sendt ham den.	Jens hasn’t sent him it.
Th-R	Passive	Den ble forært ham.	It was donated him.
Th-R	Active-OS	Erik forærte den ham ikke.	Erik didn’t donate it him.
Th-R	Active-non-OS	Erik har ikke forært den ham.	Erik hasn’t donated it him.
R-Th	Passive	Han ble forært den.	He was donated it.
R-Th	Active-OS	Erik forærte ham den ikke.	Erik didn’t donate him it.
R-Th	Active-non-OS	Erik har ikke forært ham den.	Erik hasn’t donated him it.
Th-R	Passive	Den ble rakt ham.	It was handed him.
Th-R	Active-OS	Anne rakte den ham ikke.	Anne didn’t hand it him.
Th-R	Active-non-OS	Anne har ikke rakt den ham.	Anne hasn’t handed it him.
R-Th	Passive	Han ble rakt den.	He was handed it.
R-Th	Active-OS	Anne rakte ham den ikke.	Anne didn’t hand him it.
R-Th	Active-non-OS	Anne har ikke rakt ham den.	Anne hasn’t handed him it.
Th-R	Passive	Den ble overrakt ham.	It was handed him.
Th-R	Active-OS	Kari overrakte den ham ikke.	Kari didn’t hand it him.
Th-R	Active-non-OS	Kari har ikke overrakt den ham.	Kari hasn’t handed it him.
R-Th	Passive	Han ble overrakt den.	He was handed it.
R-Th	Active-OS	Kari overrakte ham den ikke.	Kari didn’t hand him it.
R-Th	Active-non-OS	Kari har ikke overrakt ham den.	Kari hasn’t handed him it.
Th-R	Passive	Den ble lovt ham.	It was promised him.
Th-R	Active-OS	Inger lovte den ham ikke.	Inger didn’t promise it him.
Th-R	Active-non-OS	Inger har ikke lovt den ham.	Inger hasn’t promised it him.
R-Th	Passive	Han ble lovt den.	He was promised it.
R-Th	Active-OS	Inger lovte ham den ikke.	Inger didn’t promise him it.
R-Th	Active-non-OS	Inger har ikke lovt ham den.	Inger has not promised him it.
Th-R	Passive	Den ble fakset ham.	It was faxed him.
Th-R	Active-OS	Kristin fakset den ham ikke.	Kristin didn’t fax it him.
Th-R	Active-non-OS	Kristin har ikke fakset den ham.	Kristin hasn’t faxed it him.
R-Th	Passive	Han ble fakset den.	He was faxed it.
R-Th	Active-OS	Kristin fakset ham den ikke.	Kristin didn’t fax him it.
R-Th	Active-non-OS	Kristin har ikke fakset ham den.	Kristin hasn’t faxed him it.
Th-R	Passive	Den ble fortalt ham.	It was told him.
Th-R	Active-OS	Nils fortalte den ham ikke.	Nils didn’t tell it him.
Th-R	Active-non-OS	Nils har ikke fortalt den ham.	Nils hasn’t told it him.
R-Th	Passive	Han ble fortalt den.	He was told it.
R-Th	Active-OS	Nils fortalte ham den ikke.	Nils didn’t tell him it.
R-Th	Active-non-OS	Nils har ikke fortalt ham den.	Nils hasn’t told him it.
Th-R	Passive	Den ble tilbudt ham.	It was offered him.
Th-R	Active-OS	Hannah tilbød den ham ikke.	Hannah didn’t offer it him.
Th-R	Active-non-OS	Hannah har ikke tilbudt den ham.	Hannah hasn’t offered it him.
R-Th	Passive	Han ble tilbudt den.	He was offered it.
R-Th	Active-OS	Hannah tilbød ham den ikke.	Hannah didn’t offer him it.
R-Th	Active-non-OS	Hannah har ikke tilbudt ham den.	Hannah hasn’t offered him it.
Th-R	Passive	Den ble framlagt ham.	It was submitted him.
Th-R	Active-OS	Thomas framla den ham ikke.	Thomas didn’t submit it him.
Th-R	Active-non-OS	Thomas har ikke framlagt den ham.	Thomas hasn’t submitted it him.
R-Th	Passive	Han ble framlagt den.	He was submitted it.
R-Th	Active-OS	Thomas framla ham den ikke.	Thomas didn’t submit him it.
R-Th	Active-non-OS	Thomas har ikke framlagt ham den.	Thomas hasn’t submitted him it.

**Table 8:** Experimental items for Swedish survey

Order	Verb type	Swedish	English
R-Th	Monomorph	Pojken gavs priset.	The boy was given the prize.
R-Th	Bimorph	Pojken tilldelades priset.	The boy was awarded the prize.
Th-R	Monomorph	Priset gavs pojken.	The prize was given the boy.
Th-R	Bimorph	Priset tilldelades pojken.	The prize was awarded the boy.
R-Th	Monomorph	Flickan såldes bilen.	The girl was sold the car.
R-Th	Bimorph	Flickan anförtroddes bilen.	The girl was entrusted the car.
Th-R	Monomorph	Bilen såldes flickan.	The car was sold the girl.
Th-R	Bimorph	Bilen anförtroddes flickan.	The car was entrusted the girl.
R-Th	Monomorph	Gästerna visades parken.	The farmer was shown the park.
R-Th	Bimorph	Gästerna förevisades parken.	The farmer was shown the park.
Th-R	Monomorph	Parken visades gästerna.	The park was shown the farmer.
Th-R	Bimorph	Parken förevisades gästerna.	The park was shown the farmer.
R-Th	Monomorph	Mannen skickades formuläret.	The man was sent the form.
R-Th	Bimorph	Mannen tillsändes formuläret.	The man was sent the form.
Th-R	Monomorph	Formuläret skickades mannen.	The form was sent the man.
Th-R	Bimorph	Formuläret tillsändes mannen.	The form was sent the man.
R-Th	Monomorph	Dottern lämnades bostaden.	The daughter was left the apartment.
R-Th	Bimorph	Dottern erbjöds bostaden.	The daughter that was offered the apartment.
Th-R	Monomorph	Bostaden lämnades dottern.	The apartment was left the daughter.
Th-R	Bimorph	Bostaden erbjöds dottern.	The apartment was offered the daughter.
R-Th	Monomorph	Grevinnan skänktes gåvan.	The countess was given the gift.
R-Th	Bimorph	Grevinnan förärades gåvan.	The countess was presented the gift.
Th-R	Monomorph	Gåvan skänktes grevinnan.	The gift was given the countess.
Th-R	Bimorph	Gåvan förärades grevinnan.	The gift was presented the countess.
R-Th	Monomorph	Prinsen lovades makten.	The prince was promised the power.
R-Th	Bimorph	Prinsen beviljades makten.	The prince was granted the power.
Th-R	Monomorph	Makten lovades prinsen.	The power was promised the prince.
Th-R	Bimorph	Makten beviljades prinsen.	The power was granted the prince.
R-Th	Monomorph	Chefen sändes planen.	The boss was emailed the plan.
R-Th	Bimorph	Chefen förelades planen.	The boss was submitted the plan.
Th-R	Monomorph	Planen sändes chefen.	The plan was sent the boss.
Th-R	Bimorph	Planen förelades chefen.	The plan was submitted the boss.
R-Th	Monomorph	Fiskaren lånades båten.	The fisher was lent the boat.
R-Th	Bimorph	Fiskaren tillställdes båten.	The fisher was handed over the boat.
Th-R	Monomorph	Båten lånades fiskaren.	The boat was lent the fisher.
Th-R	Bimorph	Båten tillställdes fiskaren.	The boat was handed over to the fisher.
R-Th	Monomorph	Barnet räcktes boken.	The child was handed the book.
R-Th	Bimorph	Barnet överräcktes boken.	The child was handed over the book.
Th-R	Monomorph	Boken räcktes barnet.	The book was handed the child.
Th-R	Bimorph	Boken överräcktes barnet.	The book was handed over to the child.
R-Th	Monomorph	Kvinnan lärdes dikten.	The woman was taught the poem.
R-Th	Bimorph	Kvinnan tillskrevs dikten.	The woman was attributed the poem.
Th-R	Monomorph	Dikten lärdes kvinnan.	The poem was taught the woman.
Th-R	Bimorph	Dikten tillskrevs kvinnan.	The poem was attributed the woman.
R-Th	Monomorph	Eleven ordnades jobbet.	The student was arranged the job.
R-Th	Bimorph	Eleven förespeglades jobbet.	The student was held out the prospect of the job.
Th-R	Monomorph	Jobbet ordnades eleven.	The job was arranged for the student.
Th-R	Bimorph	Jobbet förespeglades eleven.	The prospect of the job was held out for the student.

## Footnotes

1 The *t*-tests use degrees of freedom approximated using the Satterthwaite method in the lmerTest package (Kuznetsova et al. 2016).

2 No regional or sex effects emerged as significant in the modeling. The results revealed an age effect with younger speakers favoring R-Th orders in passives and older speakers favoring Th-R orders. See Haddican et al. (2016) for a discussion of these results, which are not directly relevant to the issues considered here.

3 This would correspond to what is called “secundative alignment” in the typological literature, as opposed to “indirective alignment” described in the text below (Malchukov et al. 2010). In our model overt case-marking is not a defining characteristic of the two alignment types, though.

4 In Kroch’s (1989) framework, grammar competition implies change. See Haddican et al. (2016) for a discussion of age effects on acceptability of theme vs. recipient passives.

5 On the basis of fieldwork with speakers from areas in Norway where dative case is particularly prevalent, Eyþórsson et al. (2012) show that none of them accept preservation of dative under passivization of the recipient of the DOC. This does not tell us whether the case is inherent or not since there are other, independent reasons why dative subjects are not allowed by the grammar of today’s Norwegian; see Holmberg and Platzack (1995: 112–115); Holmberg (2010).

6 A reviewer for *NLLT* suggests that speakers of Norwegian dialects with dative case would be expected to just have a grammar with (14), rather than the competition between grammars with (13) and (14), respectively, that most Norwegians have. Given the weak status of dative case in the dialects in question, demonstrated by Eyþórsson et al. (2012), this is somewhat unlikely. There may well be no speakers left that use dative consistently, in the relevant contexts. Our analysis revealed no preference for theme-passives among speakers who reported a hometown within the traditional dative-preserving dialect area.

7 We note that Richards’ (2004) approach to order preservation will not yield the correct facts for Norwegian, since on this approach, precedence is determined by sisterhood relations. Since Th and R are not plausibly in a sisterhood relation (nor mediated by a phonologically overt head) the proposal won’t ensure R, Th order preservation.

8 There is some variation across dialects in the representation of strings like (25b). In some dialects they behave like double object constructions on standard diagnostics, while in others, they behave like prepositional datives with a silent preposition. See Biggs (2014, 2015); Haddican (2010) and Myler (2011, 2013) for discussion. Haddican and Holmberg (2012) focus on the dialects relevant for our purposes—those where such forms behave like true DOCs.

9 Thanks to Björn Lundquist for a discussion of these data. A reviewer for *NLLT* suggests that the bad recipient passives with *ge* ‘give’ may be because of morphological blocking by the verb *få* ‘get, receive’. That is, (i) would block (ii).

However, as Norwegian has the verb *få* with exactly the same meaning and use as Swedish (main verb) *få*, how come there is no blocking in Norwegian? (Swedish also has a modal auxiliary *få* ‘may’, absent in Norwegian, but this is irrelevant.)

Furthermore, why are R-passives with other Swedish ditransitives such as *visa* ‘show’ and *skicka* ‘send’ not good, either (as our investigation, reported below, confirmed)? These verbs should not be blocked by *få* ‘get’.

10 The experiment included an additional subdesign focusing on clause type effects on object order. Each list contained an additional eight sentences from this condition. We do not discuss these results here.

11 As in the Norwegian modeling, the *t*-tests are based on degrees of freedom approximated using the Satterthwaite method in the lmerTest package (Kuznetsova et al. 2016).

12 A reviewer notes that the monomorphemic verbs generally have an alternative realization, namely the prepositional dative construction (V-DP-PP), which the bimorphemic verbs often do not have. As implied by the reviewer, this may be part of the explanation for their different status in passives. We will have to leave this suggestion for future research. A factor to take into account, in that case, is that some of the bimorphemic verbs do allow a prepositional dative alternative. For instance *tillskriva* ‘ascribe’ does not, but *förevisa* ‘show’ does.

13 Modeling revealed no effect for subject sex, age or region for either subdesign.

14 A referee for *NLLT* suggests that the relevant bimorphemic verbs may be derived by preposition incorporation in the syntax. This is an attractive idea as it would, presumably, simplify case assignment, but there are two related reasons to reject it. First, only some of the bimorphemic verbs have a counterpart with a bare verb and a PP. *Tillsända* ‘send’ and (arguably) *förevisa* ‘show’ do, but for example *tilldela* ‘award’ and *anförtro* ‘entrust’ do not. Second, with the possible exception of (again) *tillsända* and *förevisa*, the meaning of the bimorphemic verbs is not compositionally derived from the meaning of the corresponding bare verb and the preposition. If compositional meaning is a criterion, then the bimorphemic verbs are not derived by incorporation.

15 A reviewer for *NLLT* points out that some of the verbs with prepositional prefixes can take their two objects in either order, in active predicates. If this is true across the board, this would explain why they, of all Swedish verbs, accept both R-passives and Th-passives, and furthermore, it would explain it purely on the basis of locality. The interesting observation holds true perhaps most clearly for the verb *tillskriva* ‘ascribe’.

Here the R-passive could be derived from the underlying structure of (ia), and the Th-passive from the underlying structure of (ib). This duality does not seem to hold for the other verbs in our investigation, though, not even the ones that are most amenable to R- and Th-passives. *Tilldela* ‘award’ and *förevisa* ‘show’ are two of them.

For *tilldela* the definiteness of the objects seems to have a marginal effect, but not for *förevisa*.

16 If the choice between the synthetic and the periphrastic passive makes no difference in the DOC, Danish should allow R-passives but not Th-passives, where Norwegian accepts both. While this requires more careful research, preliminary results indicate that this is right (thanks to Sten Vikner for discussion of this point). Compare the *s*-passives in Norwegian (i) with the ones in Danish (ii).
